# Molecular test for screening malaria-infected blood donors to maximise recipient safety in Acre State, a Brazilian endemic area

**DOI:** 10.1590/0074-02760240109

**Published:** 2024-12-09

**Authors:** Thereza Cristina Picado Pinheiro, Sabrina Silva Santos, Fernanda Moura El Bayet Simião, Aline Rosa de Lavigne Mello, Cinara de Barros Pimentel, Leonardo Assad Lomonaco, Patrícia Alvarez, Cláudio Tadeu Daniel-Ribeiro, Rosalina Jorge Koifman, Maria de Fátima Ferreira-da-Cruz

**Affiliations:** 1 Universidade Federal do Acre, Programa de Pós-Graduação em Saúde Coletiva, Rio Branco, AC, Brasil; 2 Fundação Oswaldo Cruz-Fiocruz, Escola Nacional de Saúde Pública Sérgio Arouca, Rio de Janeiro, RJ, Brasil; 3 Centro de Hematologia e Hemoterapia do Acre, Hemonúcleo de Cruzeiro do Sul, AC, Brasil; 4 Fundação Oswaldo Cruz-Fiocruz, Instituto Oswaldo Cruz, Laboratório de Pesquisa em Malária / Centro de Pesquisa, Diagnóstico e Treinamento em Malária, Laboratório de Referência do Ministério da Saúde para Diagnóstico de Malária na Região Extra-Amazônica, Rio de Janeiro, RJ, Brasil; 5 Centro de Hematologia e Hemoterapia do Acre, Hemoacre, Rio Branco, AC, Brasil; 6 Fundação Oswaldo Cruz-Fiocruz, Instituto de Tecnologia em Imunobiológicos, Bio-Manguinhos, Rio de Janeiro, RJ, Brasil

**Keywords:** malaria, blood donors, P. vivax, P. falciparum, malaria diagnosis

## Abstract

**BACKGROUND:**

Although blood transfusion is an essential therapeutic procedure, it can present risks, including transmitting infectious diseases, such as malaria. In Acre, the thick blood smear microscopic examination (TBS) is used to screen infected malaria blood donors. However, TBS has low sensitivity for detecting *Plasmodium* in situations of low parasitaemia, such as those presented by asymptomatic clinically healthy individuals.

**OBJECTIVES:**

To investigate the pertinence of using polymerase chain reaction (PCR) to detect malarial infection for screening blood donors in Cruzeiro do Sul, Acre, an endemic high-risk malaria area in the Legal Amazon.

**METHODS:**

A cross-sectional study was conducted among individuals eligible and ineligible to be blood donors, according to clinical and epidemiological criteria. Besides the mandatory screening of HCV, HBV, and HIV tests, malaria PCR and TBS were also carried out on all blood donor candidates who attended the Cruzeiro do Sul Blood Centre from July to September 2022.

**FINDINGS:**

Of the 230 participants, 209 (91%) were eligible for blood donation by clinical-epidemiological screening. Surprisingly, no blood donor candidate reported a history of malaria. All TBS microscopic tests were negative at the time of recruitment. However, samples from four blood donor candidates (two eligible by clinical and epidemiological malaria criteria and two ineligible by hypertension and recent tattoo) were positive by *Plasmodium* and *P. vivax* molecular tests.

**MAIN CONCLUSIONS:**

Malaria molecular techniques for screening blood donors should be introduced in the Brazilian Blood Centres to maximise recipient safety. Furthermore, selecting zero-risk donors could pave the way to build a transmissible malaria-free environment in the blood bank context in the near future.

Malaria is a vector-borne disease caused by protozoan parasites of the genus *Plasmodium*. Vectorial transmission (the most common mode of malaria transmission parasites) occurs during a blood meal from an infected female *Anopheles* mosquito. However, malaria may also spread by vertical transmission from infected mothers to their unborn babies (congenital malaria), by the use of contaminated needles or syringes, or by infected blood donors to their recipients (transfusion-transmitted malaria/TTM), including organ/tissue transplantation. Indeed, TTM is underestimated mainly in malaria-endemic areas where it may be misdiagnosed as a naturally acquired infection.

In Brazil, according to data from the Ministry of Health, there are 2,800,000 blood transfusions annually in the public assistance network and 690,000 in the private sector.^1^ Though haemotherapy is a life-sustaining modality, it continues to be a possible source of disease transmission and, therefore, haemovigilance is a matter of concern in malaria-prone-world countries. To control transfusion-transmitted disease, one of the strategies adopted in Brazil is the mandatory serological testing for human immunodeficiency virus (HIV) I/II, human T-lymphotropic virus (HTLV) I/II, hepatitis B (HB) (anti-HBc and HBsAg), hepatitis C (HC), syphilis, and Chagas disease. The serological screening is complemented with mandatory molecular tests for HCV, HBV, and HIV for every blood donation.^2^ ^,^ ^3^ ^,^ ^4^ ^,^ ^5^

Concerning malaria, the thick blood smear (TBS) is the mandatory laboratory testing for all blood donors in the so-called Amazonia Legal, where more than 99% of Brazilian malaria cases occur.^4^ ^,^ ^6^ The Amazonia Legal corresponds to 59% of the Brazilian territory. It comprises nine states (Acre, Amapá, Amazonas, Mato Grosso, Pará, Rondônia, Roraima, Tocantins and the part of the State of Maranhão west of the 44ºW meridian), totalling 5.0 million km². The malaria serological tests are not recommended in endemic areas because they cannot distinguish recent, actual, or past malaria infections.

The TBS, a microscopic test widely recommended by the Ministry of Health, is used to screen malaria infection in blood donors. However, the TBS test has limited sensitivity to detect malaria infection in low-parasitised asymptomatic *Plasmodium* reservoir individuals, which makes it undetectable in clinical screening.^6^ ^,^ ^7^ ^,^ ^8^

Over time, an extensive review evaluating transfusion malaria cases in America highlighted a gap in case identification in endemic areas, where no adequately sensitive test was available in Haemotherapy services. In these places, malaria infections due to low parasitised blood donors contribute to the transmission of the disease, showing the relevance of screening blood donors with more sensitive molecular tests, especially in areas that are moving towards eliminating the disease.^9^

In this way, this study aims to investigate the feasibility of replacing TBS with polymerase chain reaction (PCR) to screen blood donor candidates in the Blood Centres of the Brazilian malaria-endemic area.

## SUBJECTS AND METHODS

*Casuistic* - This is a cross-sectional descriptive study with prospective data collection, carried out with candidates for blood donation at the Cruzeiro do Sul Blood Centre, Acre, from July 18th to September 18th, 2022. Cruzeiro do Sul, where the regional public Blood Centre is located, is the second most developed municipality in Acre State and the only place for blood donation in the Juruá region (Figure). All blood donor candidates are volunteers, and the donations are not reimbursed in public Blood Centres.

**Figure:**
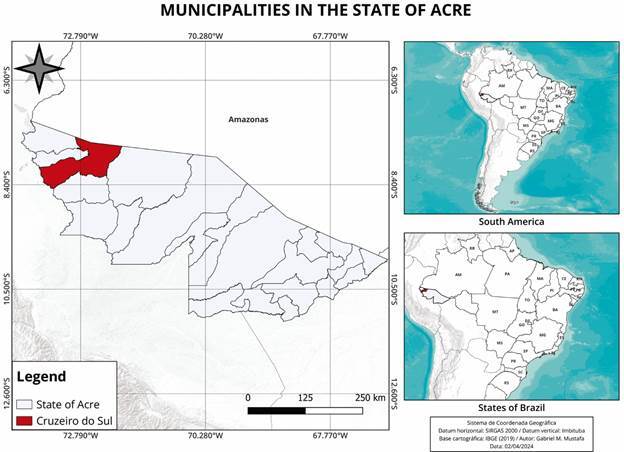
Municipalities of the Brazilian Acre State.

Concerning malaria screening, as standardised in the Service, blood donor candidates underwent clinical and epidemiological questionnaires when they were also asked about their intention to participate in the study, regardless of the result of this screening (eligible or not). From those who agreed to participate in the study and signed the informed consent, blood samples (finger prick and whole blood) were collected. Then, the blood of volunteer participants was submitted to the TBS *Plasmodium* identification microscopic test and PCRs.

For microscopic examination, thick blood smears were examined by a technician experienced in malaria diagnosis to identify malaria parasites. Thick blood smears from all the subjects were stained with Giemsa, and 100 microscopic fields were examined under a 1000-fold magnification. There was no interobserver variability since the same professional evaluated all slides.

*Sample collection, processing, and storage procedure* - Whole blood samples were collected in a tube containing anticoagulant (K2EDTA BD Vacutainer PPT) and centrifuged for 10 min at 1,300 x *g*. Buffered glycerol solution (3.024% of sorbitol, 0.648% NaCl, and 3.57% glycerol in distilled water) was added v/v to the blood pellet. Samples were gently shaken for resuspension and immediately stored at -20ºC. Subsequently, for molecular testing, by real-time (RT-PCR) and conventional PCRs, these samples were transported by air, following all the required feasibility and safety criteria, to the Laboratory of Malaria Research (Fiocruz), the headquarters of the Centre for Malaria Research and Training, a reference centre for malaria diagnosis in the Extra-Amazonian Region for the Brazilian Ministry of Health.

*PCR procedures* - Genomic DNA was extracted from 1 mL of whole blood using QIAamp midi columns as described by the manufacturer (Qiagen^®^). Malaria diagnosis was initially performed by qualitative *Plasmodium*-specific real-time PCR capable of detecting at least 0.05 parasite/μL.^10^ Then, the *Plasmodium*-positive samples were subjected to qualitative real-time PCR to *P. vivax* with a LoD of 0.01 parasite/μL and to nested PCRs for *P. falciparum* and for *P. malariae*, allowing the detection of 0.005 parasites/μL. All the tests were done in triplicate and repeated twice.^11^ ^,^ ^12^ ^,^ ^13^

*Ethical aspects* - The Federal University of Acre Research Ethics Committee approved the study under number 3,989,886 on April 24, 2020 (CAEE: 24058719.0.0000.5010), and all participants signed the Informed Consent Form.

## RESULTS

During the analysed period, there were 346 candidates for blood donation at the Cruzeiro do Sul Blood Centre. Among them, 230 were accepted to participate and were included in the study. Of the 230 donors, 94 (41%) were first-time donors: 12(13%) were from individuals considered ineligible to donate, and 82 (87%) were from eligible blood donors. The positive PCR malaria cases were from first-time donors.

Participants were 29,5 years of mean age, ranging from 16 to 68, mostly male (59%) and single (78%), and 38% had complete High School degrees (Table). Of them, 9% (21) were not considered eligible for blood donation by the clinical screening due to anaemia, ongoing or recent antibiotic therapy, cardiovascular changes, pre-existing neurological disease, tattoos, low haematocrit, and cigarette use.

**TABLE:** Sociodemographic data of blood donor candidates at the Cruzeiro do Sul Blood Centre from July to September 2022

Variables	N	%
Gender		
Male	135	58.7
Female	95	41.3
Age range (years)		
16 - 30	143	62.2
31 - 55	81	35.2
56 - 70	6	2.6
Marital status		
Single	180	78.3
Married	45	19.6
Others	5	2.2
Education		
No Education	3	1.3
Incomplete Middle School	13	5.7
Complete Middle School	5	2.2
Incomplete High School	43	18.7
Complete High School	88	38.3
Incomplete University	46	20.0
Complete University	32	13.9
Total	230	

No candidate was considered ineligible to donation due to malaria-related reasons since none reported a history of malaria in the last year, and all microscopic examinations were negative for *Plasmodium*. Therefore, according to malaria exclusion criteria, all the blood donor candidates were eligible to donate. *Plasmodium* detection by PCR resulted in four (1.7%) *P. vivax* positive samples - two of the 209 eligible (0.96%) and two of the 21 ineligible blood donors (9.5%) - showing Ct values of 35.5 and 34.3 for *P. vivax* and 31.7 and 29.8 for *Plasmodium*, respectively.

## DISCUSSION

The present study evaluates the pertinence of molecular tests, compared to microscopic ones, for *Plasmodium* detection in 230 blood donor candidates at the Cruzeiro do Sul Blood Centre in the Brazilian Amazon. Prevention of malaria transfusion disease is challenging, especially in endemic areas. Epidemiological, clinical, or laboratory identification of infected *Plasmodium* blood donors can be arduous in these regions since individuals residing in these localities can acquire a certain degree of immunity after repeated exposure to the parasite, generating asymptomatic infections.^14^ ^,^ ^15^ ^,^ ^16^ These individuals have low parasitaemia, which was not detected by the TBS microscopic examination commonly recommended in Brazilian Haemotherapy services located in endemic malaria regions.

In the extensive Brazilian territorial area, two distinct epidemiological scenarios for malaria are presented: an area considered endemic, the Amazon Region, and a non-endemic one, corresponding to the rest of the country that comprises 17 out of the 26 states and the Federal District of the country.^17^ ^,^ ^18^ The Brazilian legislation adopts different clinical, epidemiological, and laboratory malaria screening criteria in these two scenarios. In an endemic area, according to Consolidation Ordinance number 5 of 2017, which approves the technical regulation of chemotherapy procedures, clinical and epidemiological screening must address questions to candidate donors about their previous history of malaria and travel to places where malaria is endemic. Thus, blood donor candidates with a history of malaria in the last year or past *P. malariae* infection cannot donate blood at any time. In addition, the Consolidation Ordinance requires tests to detect *Plasmodium* or plasmodial antigens in all units of donated blood.^4^

Cruzeiro do Sul and the neighbouring municipalities have high endemicity for malaria.^19^ ^,^ ^20^ ^,^ ^21^ The Blood Centre of Cruzeiro do Sul is this municipality’s only blood donor collection facility and receives individuals from all the adjacent endemic municipalities. Based on this malaria scenario of high-risk annual parasitic incidence (API), epidemiological criterium alone could lead to an important loss of blood donors, negatively impacting the stock of blood components. On the other hand, *P. vivax* can remain in infected individuals for periods longer than the 12 months stipulated by regulatory standards.^22^ Even clinical criteria alone are not safe in preventing malaria transmission due to the asymptomatic parasite carriers. Therefore, clinical and epidemiological criteria are insufficient for blood transfusion safety.

The TBS microscopic examination is adopted at Cruzeiro do Sul Blood Centre, as recommended for screening blood donor candidates in malaria-endemic areas. However, the results can vary according to the microscopist, and it is time-consuming, mainly facing negative results when an additional 100 field readings are required; thus, it is not the method of choice for diagnosing a large number of samples. Besides, false negative results can occur due to the possibility of submicroscopic parasitaemia. In fact, in this survey, *P. vivax* infection was detected by PCR but not by microscopic examination in four blood donation candidates. Among them, two were prospective blood donors deemed eligible to donate based on clinical and malaria criteria. Since both tested positive for anti-Hbc, this fortuitous finding avoided transmitting two *P. vivax*-infected 500 mL blood bags. Considering that the transmission of malaria can occur through any blood component, more recipients than those who would receive their red blood cell concentrates could be infected if their blood had been transfused.^16^

*Plasmodium vivax* was the species identified in all positive samples. This finding is unsurprising, as this is the most common parasite species outside the African continent, the predominant one in Latin America and the primary etiologic agent of malaria infection in Brazil.^23^ Unlike *P. falciparum*, *P. vivax* infection usually does not progress to a severe clinical form. However, *P. vivax* represents an even more significant challenge than *P. falciparum* for the control and elimination of the disease due to the presence of hypnozoite forms, which remain ‘dormant’ in the liver for a long time, and to the early appearance of gametocytes ― the infective mosquitoes forms.

According to our results, microscopic examination may not guarantee the desired security despite its lower cost than PCR. It may constitute a waste of time and public resources.

As a national policy, molecular testing should be the universal gold standard assay for malaria blood donor screening. The barriers to adopting it in low - and middle-income countries, like Brazil, are relative to political priorities. Studies, such as the one here present, can help draw attention to the importance of carrying out more sensitive tests than microscopic examination for blood donor screening, which will benefit the individual and the community, in addition to contributing to the reduction of transmission, notably in the era of malaria elimination in Brazil.

The use of molecular techniques for screening blood donors is already a reality throughout Brazil due to their high sensitivity, and the possibility of testing sample pools contributes to reducing costs. However, its use is mandatory for HIV, HCV, and HBV but not for *Plasmodium*, even in endemic areas.

This study demonstrates the need to adopt the more sensitive molecular tests for detecting *Plasmodium* in blood donors. The NATPlusÔ kit developed in Brazil to screen blood donors was approved by Brazilian Health Regulatory Agency (ANVISA) (# 80142170056, March 21, 2022) and included malaria in the already existing mandatory HIV, HCV, and HBV tests.^8^ This new platform was introduced in 2023 and comprises 13 testing sites; two were in malaria-endemic areas (Amazonas and Pará). However, the clinical specimen tested is plasma and not whole blood, which could lead to false negative results, mainly in asymptomatic individuals with submicroscopic parasitaemia since *Plasmodium* parasitises the erythrocytes. More studies should be conducted to evaluate the performance of NATPlusÔ compared to whole blood PCR to screen blood donors for increasing transfusion safety and malaria control.

In conclusion, malaria molecular tests must be mandatory and included in the existing blood bank legislation as national police.
